# Evaluating crop yield prediction models in illinois using aquacrop, semi-physical model and artificial neural networks

**DOI:** 10.1038/s41598-025-13453-x

**Published:** 2025-07-28

**Authors:** Vishal Gautam, Abdul Gani, Shray Pathak, Anoop Kumar Shukla

**Affiliations:** 1 Department of Civil Engineering, Indian Institute of Technology Ropar, Rupnagar, 140001 Punjab India; 2 Department of Civil Engineering, Netaji Subhas University of Technology, New Delhi, 110073 India; 3 Manipal School of Architecture and Planning, Manipal Academy of Higher Education, Manipal, 576104 Karnataka India

**Keywords:** *AquaCrop*, *Crop modelling*, *Illinois*, *Remote sensing*, *Yield*, Sustainability, Environmental sciences, Environmental social sciences, Agroecology, Civil engineering

## Abstract

Crop yield is important for agricultural productivity and the country’s economy. While crop yield estimation is an essential aspect of modern agriculture, it continues to be one of the most challenging tasks to manage effectively. Corn and soybean are the important crops in Illinois, USA, considerably enhancing the region’s agricultural output and economy. The present study integrates semi-physical model, AquaCrop and Artificial Neural Network (ANN) Models for estimating corn and soybean yields. Data of different meteorological parameters including precipitation, maximum and minimum temperature, relative humidity, wind speed, solar radiation, photosynthetically active radiation and fraction of photosynthetically active radiation, land surface water index were collected for a period of 25 years from 2000 to 2024 from NASA POWER, USDA and NASS. The observed yield of soybean and corn was ranges from 2.49 to 4.37 ton/ha and 7.06 to 14.66 ton/ha. The predicted corn yield using the AquaCrop, semi-physical, and ANN models ranged from 7.60 to 14.42 ton/ha, 9.01 to 13.42 ton/ha, and 6.81 to 15.63 ton/ha, respectively. For soybean, the predicted yield ranged from 2.80 to 4.34 ton/ha, 2.92 to 3.84 ton/ha, and 2.45 to 4.43 ton/ha, respectively. The ANN model achieves the highest coefficient of determination (R² = 0.96) in predicting soybean yield, while the semi-physical model records the lowest R² value of 0.42, indicating the superior predictive capability of the ANN model. For both corn and soybean yields, the ANN model showed the highest prediction accuracy among the other models. Thus, the study underscores the significance of employing the ANN model for crop yield estimation, particularly in the regions that share similar physiographic and meteorological conditions with Illinois.

## Introduction

According to the United Nations Food and Agriculture Organization^1^, there were around 1.23 billion people employed in the world’s agri-food systems in the year 2019. Crop production prediction is one of the major components of agricultural planning, food security strategy development, and economic forecasting. Agriculture is one of the pillars of economy of the United States of America (USA) and worldwide food supply. Yield forecasting is significantly important in guiding trade strategies, policy decisions, and resource distribution^2^. The varying climatic conditions across states, diverse soil types and agricultural practices present challenges and opportunities for crop yield modelling. Climate change, with its associated increase in weather variability has raised higher demands for solid yield prediction models. Extreme events are bound to occur more frequently, and models must modify and incorporate these nonlinear effects on crop productivity^3^. The major factors of agricultural crop production include temperature, rainfall, pH, and nutrient content present in soil, as well as irrigation water in the form of surface and groundwater. Additionally, agronomic factors like crop management practices, technology use, and plant protection measures significantly influence productivity^4–6^.

Crop development and growth monitoring, and yield prediction are the key components of agricultural planning and management^7,8^. Numerous techniques are employed in this technological age to estimate crop yields. The most frequently utilized of these are statistical techniques^9,10^, crop simulation models, and remote sensing^11^. These methods are also employed to determine how the climate affects the production of various crop varieties^12,13^. Various annual crops, including wheat, soybean, corn, sugarcane, and others, have predicted their yields using machine learning techniques.

This has led to a growing interest in crop modeling, driven by the pressing need to address global food security amid challenges such as climate change, population growth, and resource constraints^14^. Advanced crop production prediction models have been developed as a result of recent advancements in data gathering instruments, increased processing capacity, and creative modelling approaches. Crop models have been shown to be essential tools for decision-making, evaluating how management practices and climate change/variability affect the environmental performance and productivity of alternative cropping systems, and advancing more sustainable and effective agriculture^15^. They are a less costly and quicker way to investigate the impacts of agricultural land management strategies on crop yields and the environment and determine the best management to achieve economically efficient yields^16^. Crop simulation models may be used as decision support tools to evaluate the financial and risk implications of agricultural management techniques. The modeling technique may produce results that are quite accurate for developing agricultural land management plans if the models are calibrated and validated using trustworthy observable field data^17^.

AquaCrop model is used to assess crop productivity, evapotranspiration, and water use efficiency under varying degrees of water stress, as well as to analyze crop responses to diverse irrigation strategies and water levels^18,19^. The model further evaluates yield responses to the soil moisture content, fertilization practices, soil organic amendments, and their interactions^20^. It also offers insights into canopy cover, panicle biomass, and grain yield under different irrigation and nitrogen management regimes^21^, and assesses the crop yield to climate change scenarios, accounting for changes in rainfall and temperature^22^. Additionally, AquaCrop has been employed to study crop performance under different irrigation and manure management practices at high altitudes^23^, as well as responses to water stress^24,25^. The AquaCrop model has been extensively applied in studies involving maize, rice, and wheat water balance, particularly in Asia.

According to Basso & Liu^26^, these models employ a range of methodologies, such as process-based models, advanced machine learning techniques, and traditional statistical methods. Since each model has unique benefits and drawbacks, comparing them is crucial to comprehending how applicable they are in a variety of geophysical and agricultural settings. Recent developments have created chances to integrate precision agricultural data, long-term climate forecasts, and high-resolution satellite images to increase model accuracy and geographical resolution^27^. Recent developments in crop modeling provides a validation for enhanced complexity, integration with diversified data sources, and application of computational techniques^28^. Semi-physical models simulate crop growth from a physiological processes and interaction with the environment, continually improving the dynamics of the plant soil atmosphere system. The crop models are characterized by better representations of root architecture, nutrient cycling, and crop-specific behaviours, enables more accurate simulations of crop responses to abiotic environmental stressors^29^. Meanwhile, the emergence of big data and machine learning has further transformed agricultural research^30^.

In USA, 97 million hectares area used for crop production yearly. USA is one of the biggest producers and exporters of major crops, including maize, soybean, wheat, and rice, making up 87% of the total agricultural area. USA contributed 27%, 32% and 6% of the world’s yearly production of maize, soybeans, and wheat, during the last ten years (2013–2022). In turn, the US is one of the main exporters of rice, even if its area and production are relatively minor when compared to Asian nations^31^. Table 1 depicts the average production, harvested area and average yields of various crops.

**Table 1:** Average production, harvested area and yield of maize, soybean, wheat, and rice in the USA^31^.

S. No.	Crops	Total Production (Million ton)	Harvested Area (Million Ha)	Average Yield (ton/Ha)
1.	Maize (Corn)	364.1	33.6	10.8
2.	Soybean	111.2	33.6	3.3
3.	Wheat	52.3	16.5	3.1
4.	Rice	9.1	1.1	8.4

According to the United State of Department of Agricultural^32^, the average US corn production in the US between 2021 and 2023 is mainly concentrated in the Midwest, with Iowa accounting for 18% of the national output, followed by Illinois at 16%. Nebraska and Minnesota each contribute 10%, while Indiana produces 7%. South Dakota, Wisconsin, Kansas, and Ohio each account for 4–5% of the total. Similarly, soybean production is heavily cantered in the Midwest. Illinois leads with 16% of national output, followed by Iowa at 14%, Minnesota at 8%, and Indiana and Ohio each contributing 8%. Nebraska and Missouri add 6% each, while Arkansas and Mississippi, located in the southern region, each contribute a smaller share of 3%^29^.

The present study is design with the objectives (i) to estimate the corn and soybean yield in Illinois, USA by using AquaCrop, semi-physical and ANN models; (ii) comparison of the performance of these models for predicting corn and soybeans yields, and therefore identifying which model is most effective in predicting corn and soybean yield.

## Materials and methods

### Study area

**Fig. 1:**
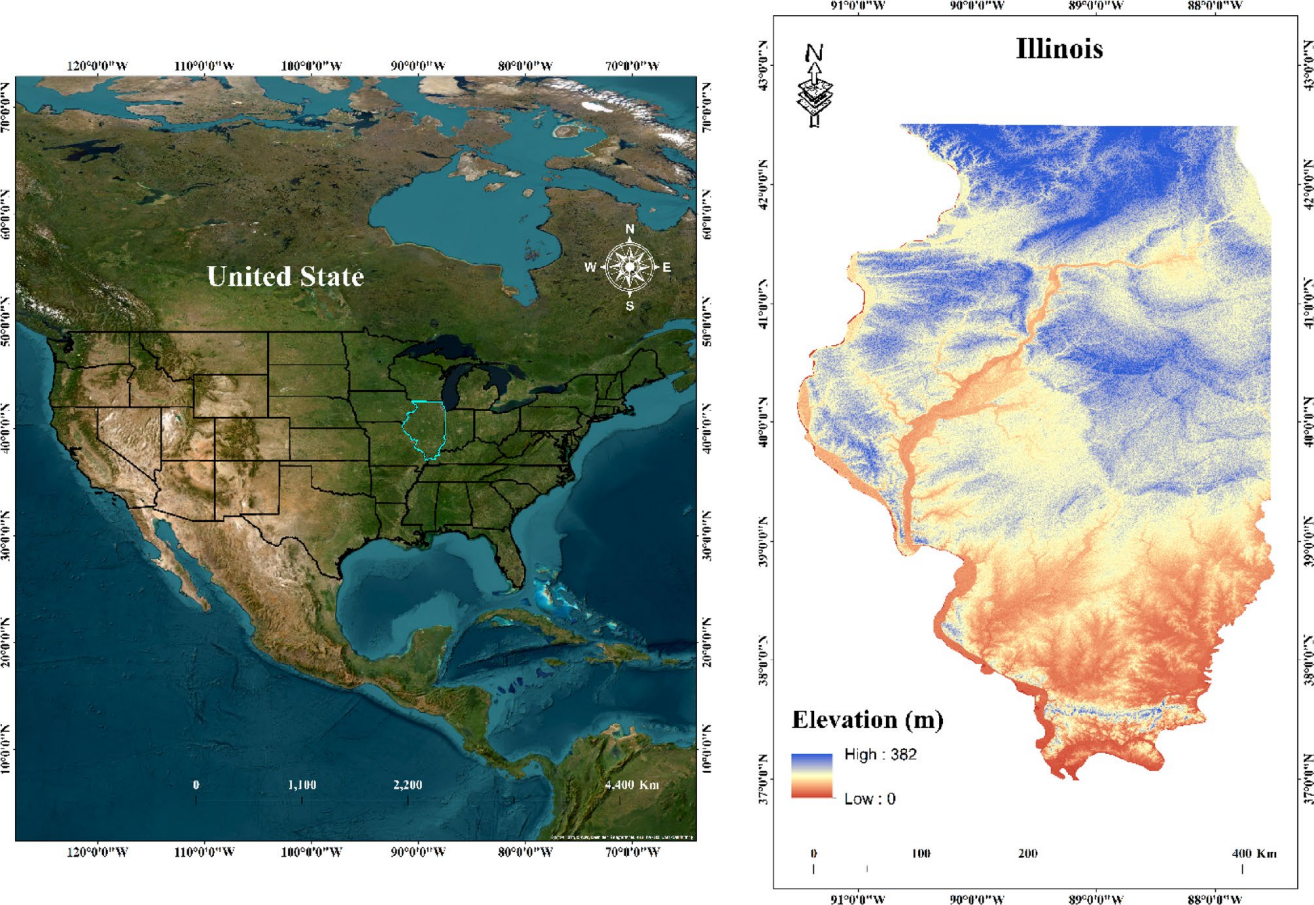
Study Area Map (created using QGIS software, version 3.40.7; available at https://qgis.org/download/).

Illinois state located in the middle of the US with significant historical, natural, and economic significance and lies in between 37° to 42°N and 87° to 91°W^33^. The state shares borders with Indiana to the East, Kentucky to the South, Iowa and Missouri to the West, and Wisconsin to the North. According to the USDA, Illinois accounts for 16% of the country’s corn production and 16% of its soybean production. These statistics highlight Illinois vital role in the national agricultural sector. Illinois is known for its diverse geography, which ranges from hills and forests in the South to flat grasslands in the Centre and North. In Illinois, average yearly temperatures range from 9 °C in the North to 14 °C in the South. Seasonal highs vary from 14 °C to 19 °C during spring and fall, while summer highs average 27 °C. Annual precipitation exceeds 48 inches in Southern Illinois, dropping to less than 32 inches in the North. Snowfall patterns are reversed, with Northern regions receiving an average of 36 inches annually, compared to less than 10 inches in the South. The Chicago area sees the most significant winter snowfall, heavily influenced by lake-effect snow from Lake Michigan. Figure 1 shows the study area of Illinois and its county boundaries.

### Data collection

The data of various parameters used in the AquaCrop, semi-physical and ANN models were collected for a period of 25 years, from 2000 to 2024, in Illinois. Daily Data of temperature, precipitation, solar radiation, wind speed, relative humidity, surface soil-wetness, soil moisture profile and root zone soil wetness were collected from NASA POWER, while photosynthetically active radiation (PAR), fraction of absorbed photosynthetically active radiation (fAPAR) and LSWI data were extracted from Moderate Resolution Imaging Spectroradiometer (MODIS) imagery and crop data layer data was collected from USDA NASS imagery. Data of PAR and fAPAR was collected from MODIS MCD18C2 and MODIS MCD15A3H imagery respectively from the month of May to September.

### Semi physical model

**Fig. 2:**
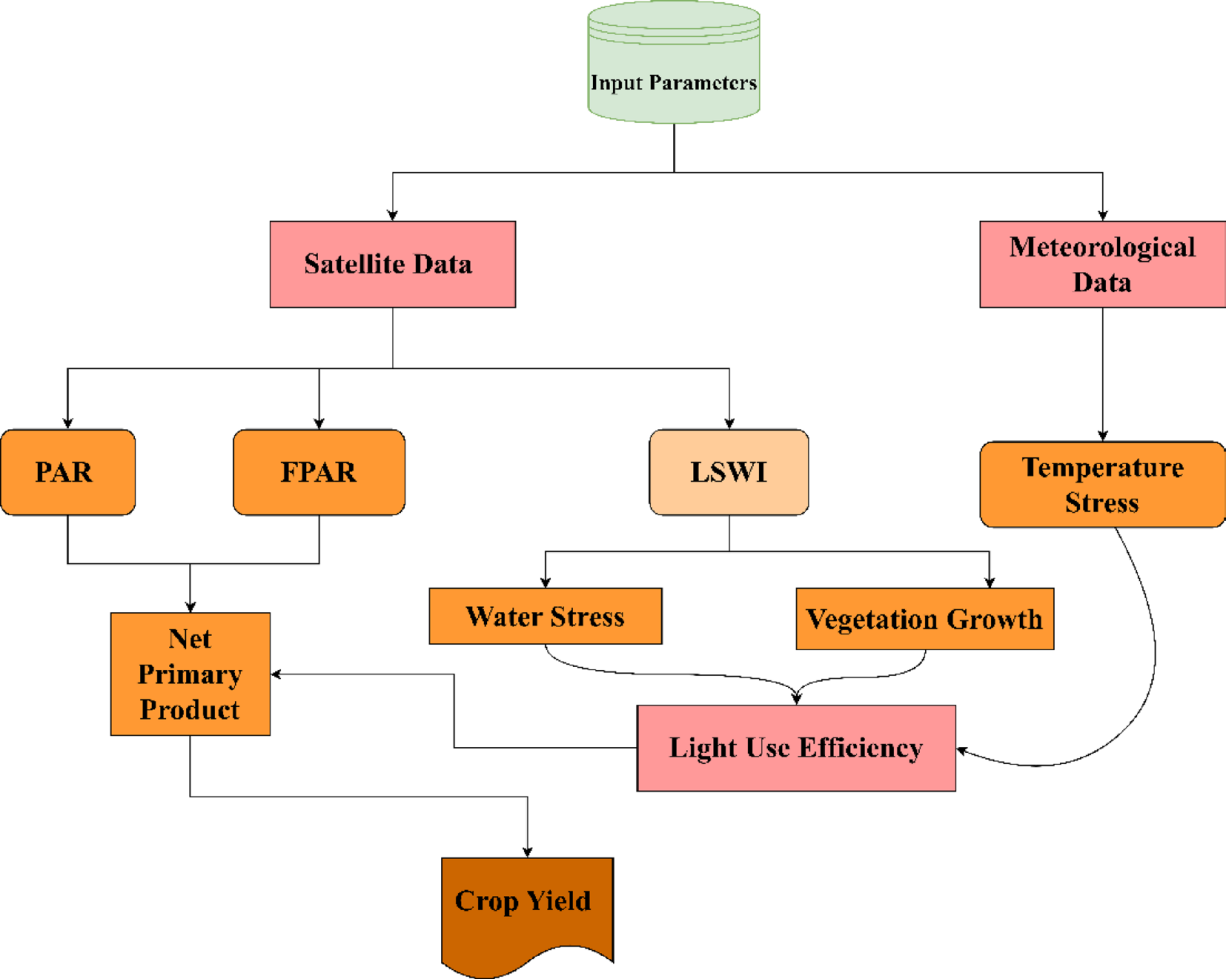
Flowchart of Semi-Physical Model.

Semi-physical models refer to the modeling technique which uses both physical and biological understanding of crop growth with empirical methods. Physical and biological processes include photosynthesis, evapotranspiration and soil-water balance whereas empirical methods include regression models or machine learning techniques to account for local factors. The semi-physical approach for assessing the corn and soybean yields combines crop growth physiology knowledge with remote sensing data to create a thorough framework for evaluating yield dynamics under various environmental circumstances. For the assessment of crop yield, PAR, fAPAR, light use efficiency (LUE), water stress, temperature stress and vegetation growth 8-day composite data were used. The model uses high resolution satellite data to estimate yield and accounts for the varied spatial distribution of environmental factors across numerous agroecological regions. Figure 2 depicts the methodology adopted for the semi-physical model.

### Light use efficiency ($\varepsilon$)

The light use efficiency (ε) is calculated by using Eq. 1^34^:

1 $$\varepsilon={\varepsilon}_{max}\times\:{T}_{s}\times\:{W}_{s}\times\:{P}_{s}$$

Where,

ℇ_max_ is the Maximum light use efficiency (gC/MJ) and its value is 0.34^35^.

W_s_ is the water stress;

T_s_ is the temperature stress;

P_s_ is the vegetation growth.

### **Water stress (W**_**s**_**)**

Remote sensing data from MOD09A1 MODIS product is used to assess water stress. Land surface water indicator (LSWI) measures moisture content in both plant and soil and calculated by using reflectance data from the shortwave infrared (SWIR) and near-infrared (NIR) bands. Using both LSWI and W_s_, water-stressed areas can be effectively identified and mapped, supporting enhanced water management strategies and improving yield predictions across various agricultural landscapes. LSWI is calculated by using the Eq. 2^36^:

2 $$\:LSWI=\frac{NIR-SWIR}{NIR+SWIR}$$

LSWI ranges from − 1 to + 1, where higher values correspond to increased moisture availability. In order to calculate W_s_ throughout the growing season, the maximum LSWI value (LSWI_max_) is determined, and water stress is calculated using the Eq. 3:

3 $$\:{W}_{s}=\frac{(1-LSWI)}{(1+{LSWI}_{max})}$$

W_s_ lies from 0 to 1, where values closer to 1 indicates higher levels of water stress.

### **Temperature stress (T**_**s**_**)**

Temperature stress during the cropping period is calculated by using Eq. 4^36^:

4 $$\:{T}_{s}=\:\frac{(T-{T}_{\text{m}\text{i}\text{n}})(T-{T}_{max})}{\left(T-{T}_{\text{m}\text{i}\text{n}}\right)\left(T-{T}_{\text{m}\text{a}\text{x}}\right)-\left(T-{T}_{opt}\right)2}$$

Where,

T is the Mean temperature (^o^C),

T_min_ is the Minimum temperature (^o^C),

T_max_ is the Maximum temperature (^o^C),

T_opt_ is the Optimum temperature for photosynthesis (^o^C) and its value is 30 °C for corn and soybeans.

### Vegetation growth

The two phases of the vegetation growth (P_s_) influence index are the leaf growth phase (P_s1_) and grain ripening phase (P_s2_) and is calculated as given by Xiao et al.^37^. The leaf growth phase is a significant phase in the crop development because it comprises of beginning and expansion of leaves, which play an important role in canopy formation and photosynthesis. This stage is crucial in determining the total amount of biomass produced, the health of the plants, and eventually the crop yield. The grain ripening phase marks the final stage of crop development and is critical for determining the harvestable yield of crops. This phase follows grain filling and is characterized by physiological maturation, moisture loss, and stabilization of seed dry matter.

5 $$\:{P}_{s1}=1\:$$

6 $$\:{P}_{s2}=\:\frac{1+LSWI\:}{2}$$

### Estimation of crop yield

Crop yield is assessed by determining net primary productivity (NPP) and NPP is calculated by using the Eq. 7^34^:

7 $$\:NPP=\:\sum\:PAR\times\:fAPAR\times\:\epsilon\:\times\:\varDelta\:t$$

After determining NPP, crop yield was further calculated by using a yield model, represented by the Eq. 8^34^:

8 $$\:Yield\:=a\times\:\sum\:NPP+b$$

Where, a and b are the regression constants for the relationship between NPP and crop yield and value of a is 0.12 and 0.073 for corn and soybean respectively, while the value of b is 0.739 and 1.298 for corn and soybean respectively as given by Na et al.^34^.

### AquaCrop model

AquaCrop is the water-driven simulation tool developed by the Food and Agriculture Organization (FAO) of the United Nation Land and Water Division for planting and scenario analysis^38,39^. The model connects the soil, crop, and atmospheric components through a comprehensive soil-water balance framework^40^. The model particularly focuses on the crop canopy, which is the upper layer of leaves and stems, to better understand how different factors influence crop development and yield in different farming environments. The model also evaluates crop yields and the associated carbon, energy, and water footprints, offering valuable insights into food production challenges and solutions to water scarcity^41^. Moreover, it supports assessments related to food security, crop productivity, sustainability, and efficiency, with a focus on water availability and consumption in agricultural production^42^.

AquaCrop model incorporated daily data of various parameters including maximum and minimum temperature, precipitation, reference evapotranspiration, initial soil water content, and soil characteristics. In the absence of ground truth data, initial parameters were employed to estimate crop yields. The growing season for corn and soybeans extended from May to September across all years. Due to variations in sowing dates across whole region, we used the average planting date of 20th May and the average harvesting date of 30th of September.

The reference evapotranspiration (ET_o_) is calculated by the modified Hargreaves method and given by Eq. 9^43^:

9 $$\:{ET}_{o}=0.0013\times\:0.408\times\:RA\times\:({T}_{avg}+17.0)\times\:{(TD-0.0123\times\:P)}^{0.76}$$

Where,

ET_o_ is the reference evapotranspiration,

RA is the extra-terrestrial radiation expressed in MJm^−2^d^−1^,

T_avg_ is the average daily temperature in ^o^C, and.

TD is the temperature range in ^o^C.

The RA is calculated as given in Eq. 10^43^:

10 $$\:RA=\:\frac{24\left(60\right)}{\pi\:}\times\:{G}_{sc}\times\:{d}_{r}\times\:[{w}_{s}\times\:\text{sin}\left(\phi\:\right)\text{sin}\delta\:+\text{cos}(\phi\:)\text{c}\text{o}\text{s}\left({\updelta\:}\right)\text{s}\text{i}\text{n}\left({w}_{s}\right)]$$

Where,

G_sc_ is the solar constant equal to 0.0820MJ m^−2^ min^−1^,

d_r_ is the inverse relative distance Earth-Sun,

δ is the solar declination in radians,

w_s_ is the sunset hour angle in radians, and.

φ is latitude in radians.

### Artificial neural network (ANN) model

**Fig. 3:**
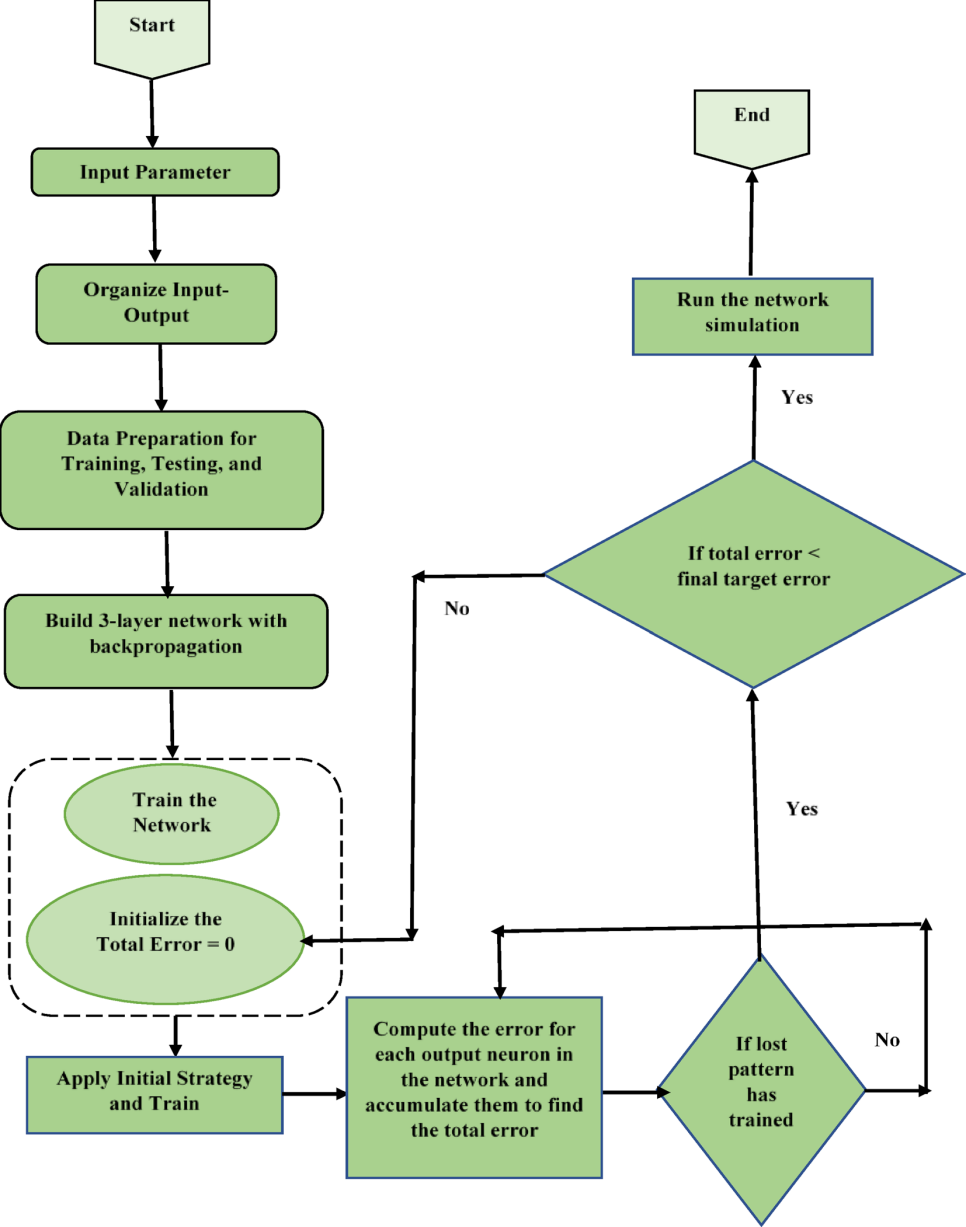
Flowchart of ANN.

Artificial neural networks (ANNs) are computational models used in machine learning that consist of interconnected processing units. By processing inputs through interconnected neurons, they handle difficult issues such as image recognition and computer vision, outperforming traditional rule-based programming^44^. ANN consists of interconnected artificial neurons that form specialized topologies such as feedforward, feedback, and lateral networks. ANN consists of three layers: input, hidden, and output. The flow diagram of the ANN process is shown in Fig. 3.

### Optimization of network architecture

The optimization of network design for crop yield prediction is an important step in developing precise and reliable agricultural prediction models. ANN provides a non-linear regression model to capture complex, non-linear correlations between input factors and crop production^45^. An input layer, one or more hidden layers, and an output layer are the usual layers of a well-structured ANN model. For developing optimal neural network, architecture for the crop yield prediction involves three important factors: (i) selection of input neurons that accurately represent relevant predictor variables, (ii) developing hidden layer configurations that demonstrate intricate nonlinear interactions, and (iii) developing output layer to effectively depict projected yield^46^. Data preprocessing is part of the optimization process for assuring that all input variables are on comparable scales and no variables dominate the learning process^47^. To assure the model’s prediction accuracy and resilience, models’ performance was evaluated by using root mean squared error (RMSE), and coefficient of determination (R²).

The ANN model was used to predict crop yield by utilizing composite data for the growing season from May to September including maximum temperature, minimum temperature, precipitation, solar radiation, wind speed, relative humidity, surface soil wetness, profile soil moisture, and root zone wetness^48^. These parameters were integral in enhancing the model’s prediction accuracy. The ANN architecture consisted of 10 hidden layers, designed to capture the complex, non-linear relationships between the input variables and crop yield. The model was trained on the 70% dataset, and its performance was assessed by using 15% datasets for each testing and validation, ensuring a robust evaluation of the model’s predictive capabilities for both corn and soybean yields.

### Performance evaluation and model selection

The performance evaluation and model selection process for ANN in crop yield prediction follows a comprehensive approach to ensure optimal results. The model’s performance is assessed using RMSE and R^2^ during the initial phase. RMSE improves interpretability by expressing errors in the same units as the crop yields, facilitating a clearer understanding of the model’s accuracy^49^. R^2^ measures how well a model predicts crop yields, with values closer to 1 indicating greater performance.

## Results and discussion

**Fig. 4:**
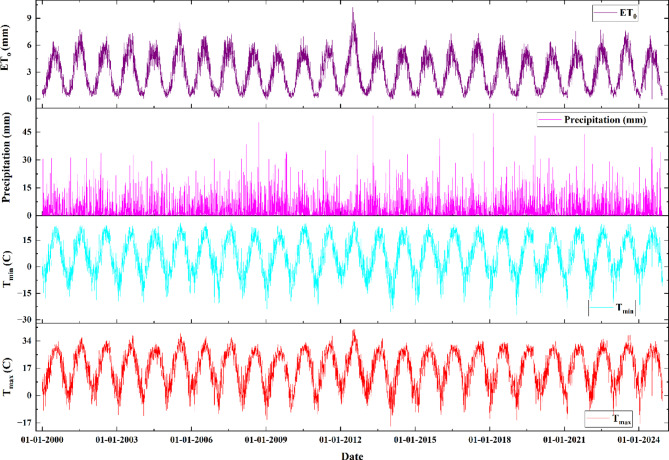
Variation of Maximum Temperature, Minimum Temperature, Precipitation and Evapo-transpiration.

Figure 4 depicts the variation of various climatic parameters including maximum temperature, minimum temperature, precipitation and evapotranspiration from the year 2000 to 2024. The maximum temperature varies from − 19.23 to 41.31 ℃ in which the highest and lowest value of maximum temperature was observed on the 4th July 2012 and 2nd January 2014. The minimum temperature varies from − 28.37 to 25.89 ℃ in which the highest and lowest value of maximum temperature was observed on the 3rd July 2012 and 26th January 2019. The daily precipitation in the study area varies from 0 to 55.15 mm, in which the maximum precipitation was observed on 16th February 2018. The reference evapotranspiration was varied in the range of 0 to 10.2 mm, in which the highest value was found on 25th June 2012.

**Fig. 5:**
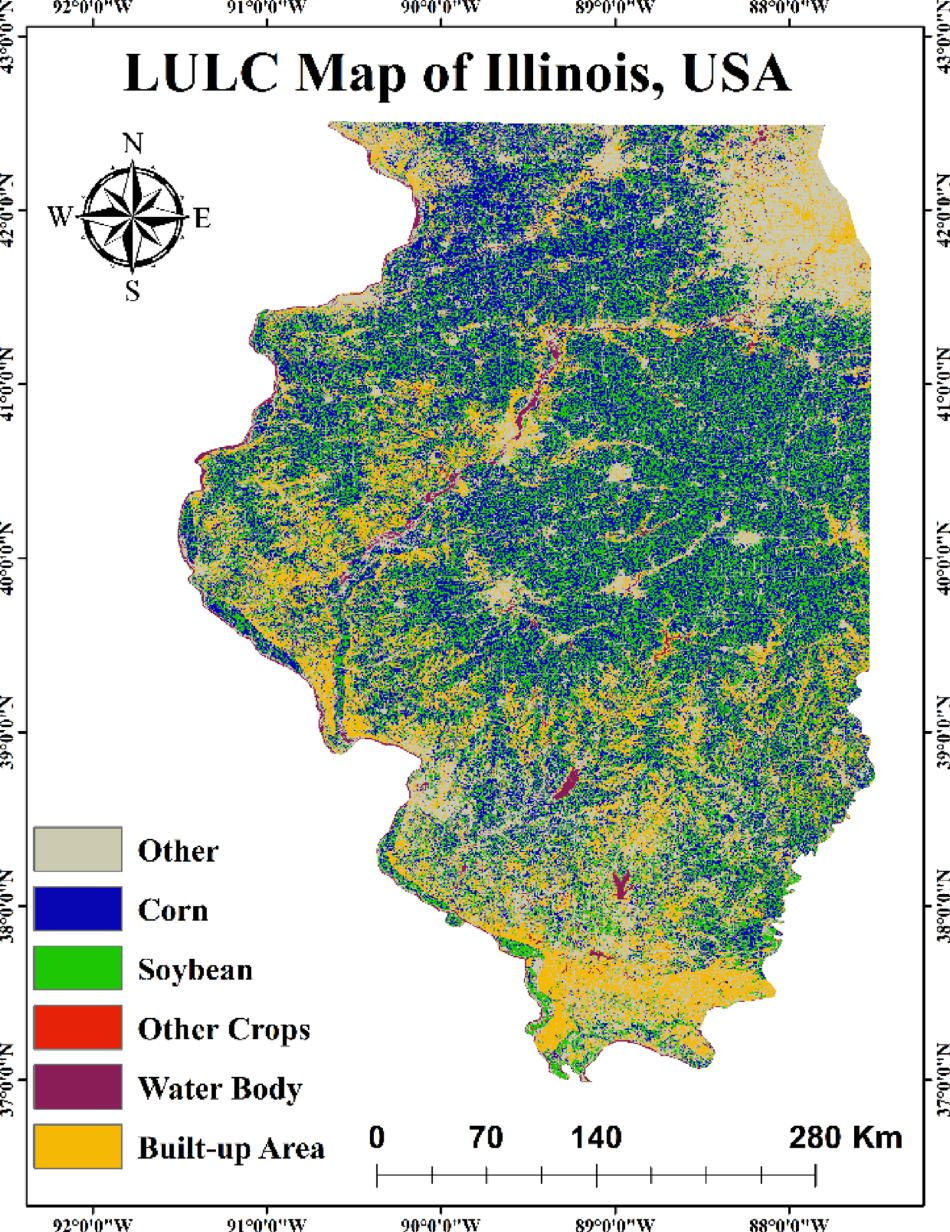
Land Use Land Cover map of Illinois USA (created using QGIS software, version 3.40.7; available at https://qgis.org/download/).

The observed yield data was collected from USDA NASS from 2000 to 2024. The yield of corn and soybeans were simulated for the growing season from May to September in Illinois, USA. The observed yield of corn and soybean varies from 7.06 ton/ha to 14.66 ton/ha and 2.49 ton/ha to 4.37 ton/ha respectively. Figure 5 depicts the land use land cover (LULC) map of Illinois, USA for the year 2023. From LULC map it has been found that corn and soybean crop area were 30.86% and 26.87% respectively. The minimum and maximum observed yield of corn was observed in 2012 and 2024 respectively, while the minimum observed yield of soybean was found in 2003 and maximum yield of soybean was found in 2021 and 2024. The yield of corn and soybean was estimated by using semi-physical model, AquaCrop model and ANN model for 25-year duration from 2000 to 2024 for Illinois, USA.

### Semi-physical model

**Fig. 6:**
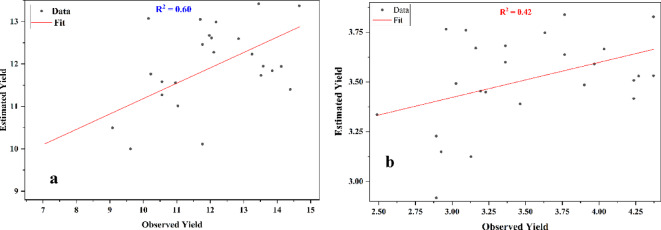
(a-b): Regression Plots of Observed and Estimated Yields by using Semi-physical Model; a: Corn and b: Soybean.

The yield of corn and soybeans was estimated for the 25-year duration from 2000 to 2024 by using the semi-physical model for the state of Illinois, USA. The assessed yield of corn varied from 9.01 to 13.42 ton/ha. The minimum yield of corn was found in 2012, whereas the maximum yield of corn was found in 2024. R^2^ value of 0.60 and RMSE value of 1.4377 ton/ha were obtained for the estimation of corn yield by using semi-physical model which indicates the model`s moderate performance of model for the corn yield estimation. The estimated yield of soybeans ranged from 2.92 ton/ha to 3.84 ton/ha. The maximum and minimum yield of soybeans were found in 2014 and 2012 respectively. R^2^ value of 0.42 and RMSE value of 0.4888 ton/ha were obtained for the estimation of soybean yield by using semi-physical model which indicates weaker performance of model for the estimation of soybean yield. Figure 6 depicts the regression plots of observed and estimated yield of corn and soybean.

**Fig. 7:**
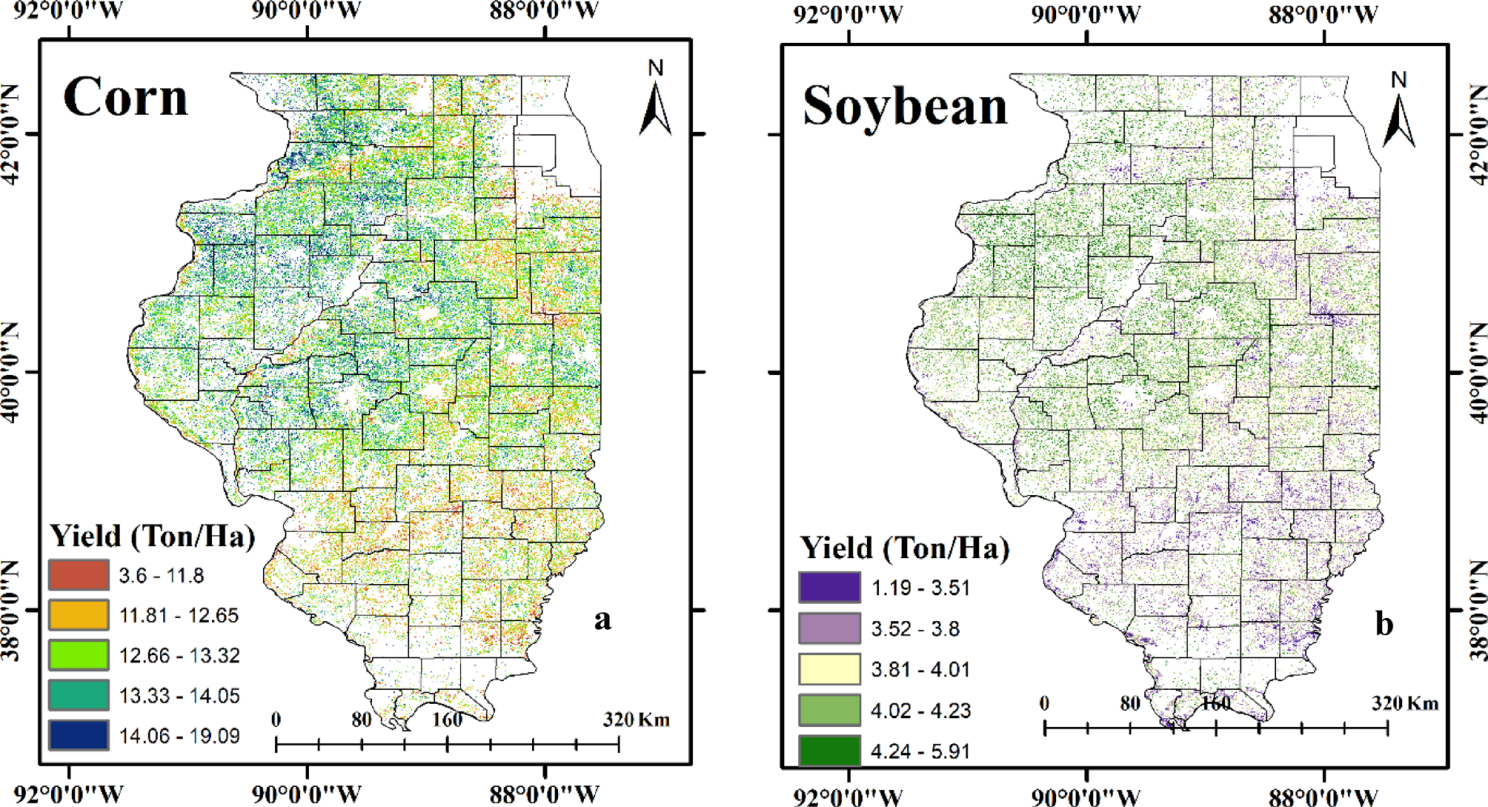
Estimated Yields in the year 2024 by using Semi-physical Model; a: Corn and b: Soybean (created using QGIS software, version 3.40.7; available at https://qgis.org/download/).

PAR and LUE are the main factors influencing the yield estimations. PAR is a significant factor in promoting photosynthesis and LUE measures how well plants convert PAR to NPP. Consequently, changes in PAR and LUE influences spatial variation in yields. A mechanistic knowledge of the variables affecting agricultural production is provided by this semi-physical method, which also offers insightful information about the complex interactions between radiation, crop physiology, and yield results. Ji et al.^50^ assessed the yield of wheat in China with R^2^ value of 0.64 and RMSE value of 0.948 ton/ha. Figure 7 shows the spatial variation of estimated yields of corn and soybean for the year 2024. The corn yield in North-East of Illinois was ranges from 11.81 ton/ha to 14.05 ton/ha, while in North-West Illinois it varies from 13.33 ton/ha to 19.09 ton/ha. Wheras in South-East Illinois corn yield ranges from 3.6 ton/ha to 13.32 ton/ha and in South-West Illinois, it ranges from 3.6 ton/ha to 19.09 ton/ha. The soybean yield in North-East and North-West of Illinois was ranges from 1.19 ton/ha to 5.91 ton/ha, while in South-East and South-West Illinois it varies from 1.19 ton/ha to 4.23 ton/ha.

### Estimation of yield using aquacrop model

The yield of corn and soybean was also estimated by using AquaCrop model which includes maximum and minimum temperature, precipitation, reference evapotranspiration, initial soil water content and soil characteristics. This study employs AquaCrop model for predicting the corn and soybean yield in the Illinois. The model uses 70% and 30% of the dataset was utilized for training and validation respectively. The model was calibrated and validated by using 18 and 7 year dataset respectively.

**Fig. 8:**
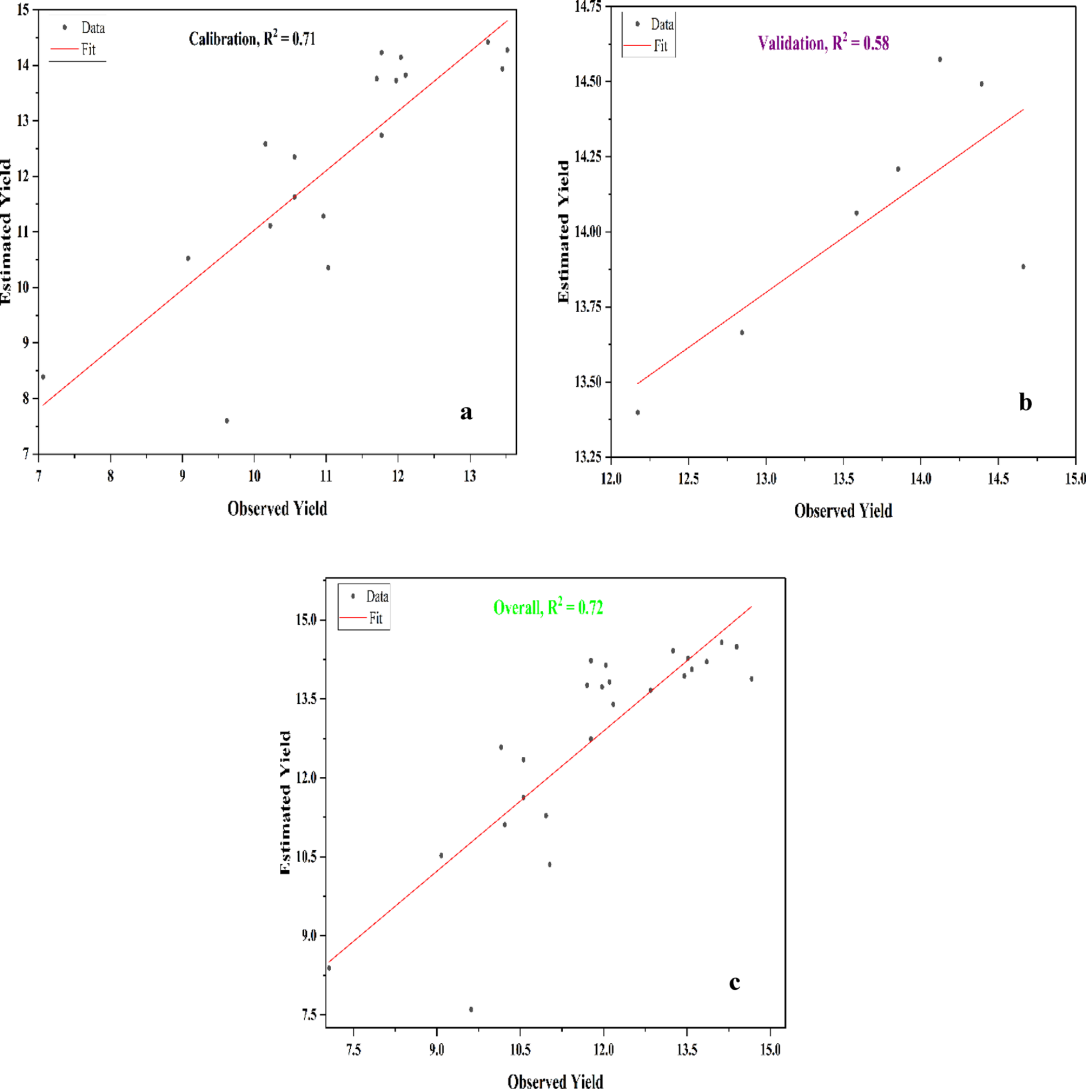
(a-c): Regression Plots of Observed and Estimated Yields of Corn by using AquaCrop Model; a: Calibration, b: Validation and c: overall.

The estimated yield of corn by using AquaCrop model ranges from 7.60 ton/ha to 14.42 ton/ha. The minimum estimated corn yield assessed in 2005, which reflects a significant deviation from the observed value, which was 9.62 ton/ha. The maximum estimated corn yield was assessed in 2024 which closely aligned with the observed yield of 14.66 ton/ha, showing the model’s accuracy in predicting higher yields in the more recent years. Figure 8 shows the regression curve of the AquaCrop model that was used in the present study to predict the corn yields during the training, and validation phases. The R^2^ value of 0.72 and RMSE value of 1.37 ton/ha were assessed for the estimation of corn yield by using AquaCrop model which indicates moderate model performance. The model achieved R^2^ values of 0.71, and 0.58 for calibration, and validation phases, respectively, indicating consistent and reliable performance across different stages. The model successfully predicted the general trends of corn yield improvement over time. Jin et al.^51^ applied the AquaCrop model to simulate maize yield and found the R^2^ value for grain yield was 0.78 and RMSE was 1.44 ton/ha in Beijing, China.

**Fig. 9:**
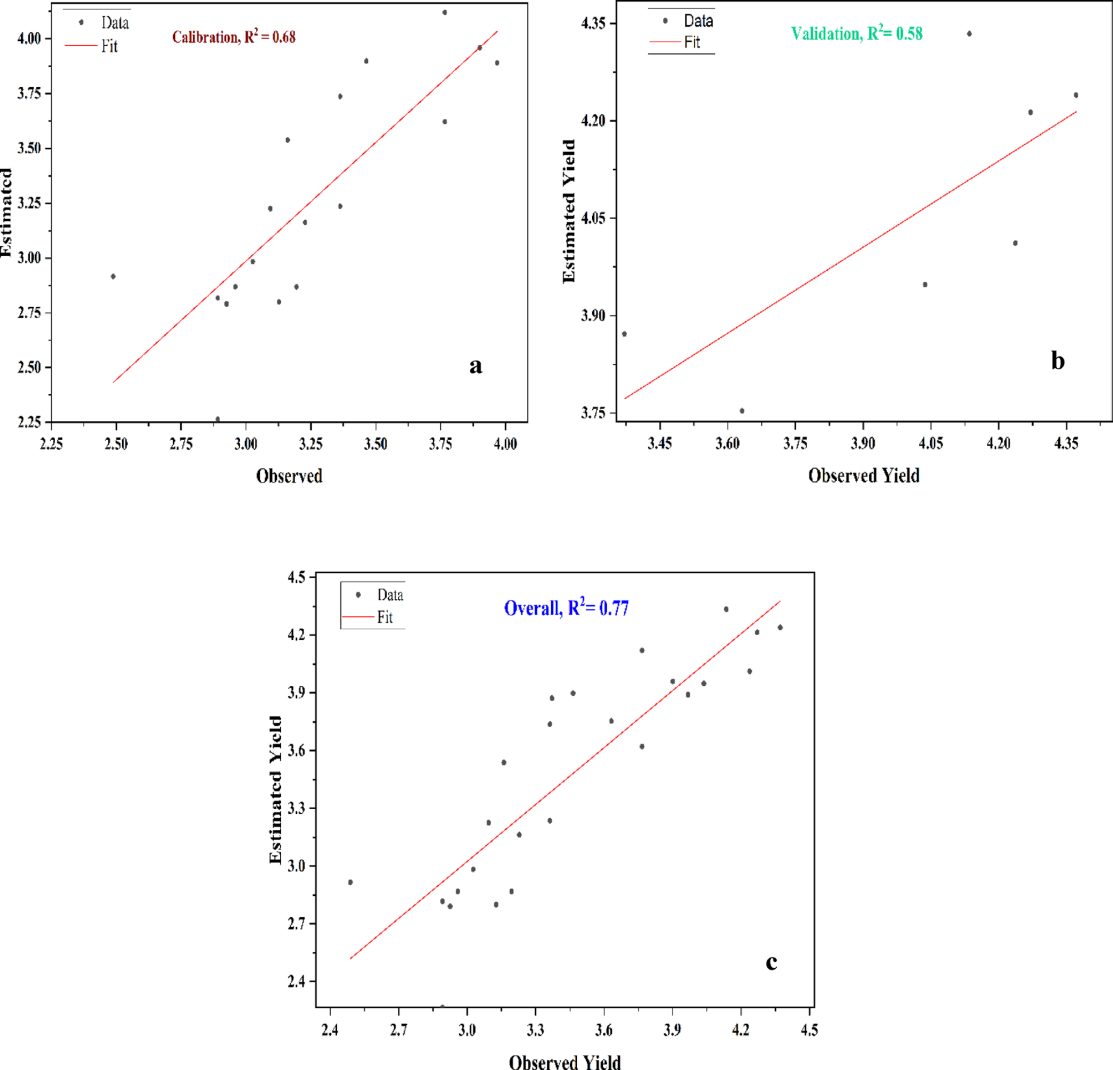
Regression Plots of Observed and Estimated Yields of Soybean by using AquaCrop Model; a: Calibration, b: Validation and c: overall.

The estimated soybean yields range from 2.80 ton/ha to 4.34 ton/ha. The minimum estimated soybean yield assessed in 2005, aligns closely with the observed yield of 3.13 ton/Ha, while the maximum estimated soybean yield in 2020 was very similar to the observed yield of 4.33 ton/Ha. This indicates that the AquaCrop model has been more effective in predicting soybean yields than corn yield in recent years. While earlier years showed some variability in estimates, AquaCrop model demonstrated improved accuracy in capturing soybean yield trends over time. Figure 9 shows the regression curve of the AquaCrop model to predict the soybean yields during the training, and validation phases. The regression plot for soybean indicates a strong correlation between predicted and observed yields, with an overall R² value of 0.77 and RMSE value of 0.29 tons per hectare, demonstrating the model’s precision. The model achieved R^2^ values of 0.68, and 0.58 for calibration, and validation phases, respectively, indicating consistent and reliable performance across different stages. These results demonstrate the model’s strong predictive capabilities, though its performance could be further improved by including additional ground truth data and relevant parameters, thereby enhancing the overall accuracy and reliability of the crop yield predictions. Adeboye et al.^52^ estimate the soybean yield in Nigeria and finds the R^2^ value of 0.99 and RMSE value of 0.03 ton/ha.

### Artificial neural network model

ANN model including one input layer, ten hidden layers, and one output layer has been employed. The z-score technique was used to standardize the input data and provide values between 0 and 1. Nine and one neurons were selected for the input and output layers, respectively. After that, the best network design and data partitioning plan were determined using the neural network fitting tools in the MATLAB 2024b program.

**Fig. 10:**
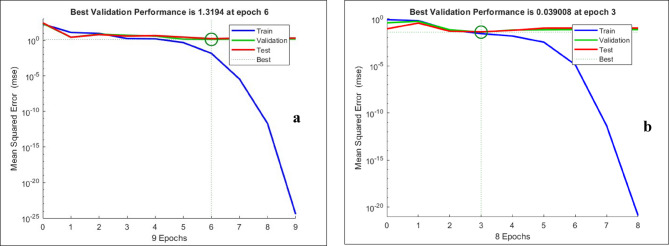
(a-b): Performance Plot for the estimation of Yield; a: Corn and b: Soybean.

This serves as the main tool for identifying optimal network architecture at a later, more thorough investigation. According to the model used in this study, 70% of the dataset was utilized for training, and 15% was used for validation. However, testing has been conducted using the remaining 15% of the dataset. The yield was then calculated using the ANN model because it works well with this partitioning approach. The training outcomes validate that the ANN model with architecture 9–10–1 is the best combination for forecasting the research area’s yield. We split the data samples for system validation testing, validation, and training in order to identify the best partitioning technique. Additionally, data was divided using a random technique that chose specific fundamental points from the whole dataset, which included data that had not been identified before. Figure 10 shows the performance plot for the estimation of corn and soybean yields. The performance plot highlights a convergence with the MSE reducing to 1.3194 at 6 epoch for the estimation of corn yield which signifies efficient learning whereas for the estimation of soybean yield, MSE decreases steadily to 0.039 at 3 epoch, which confirms the model’s capability to adapt and capture soybean yield unpredictability.

**Fig. 11:**
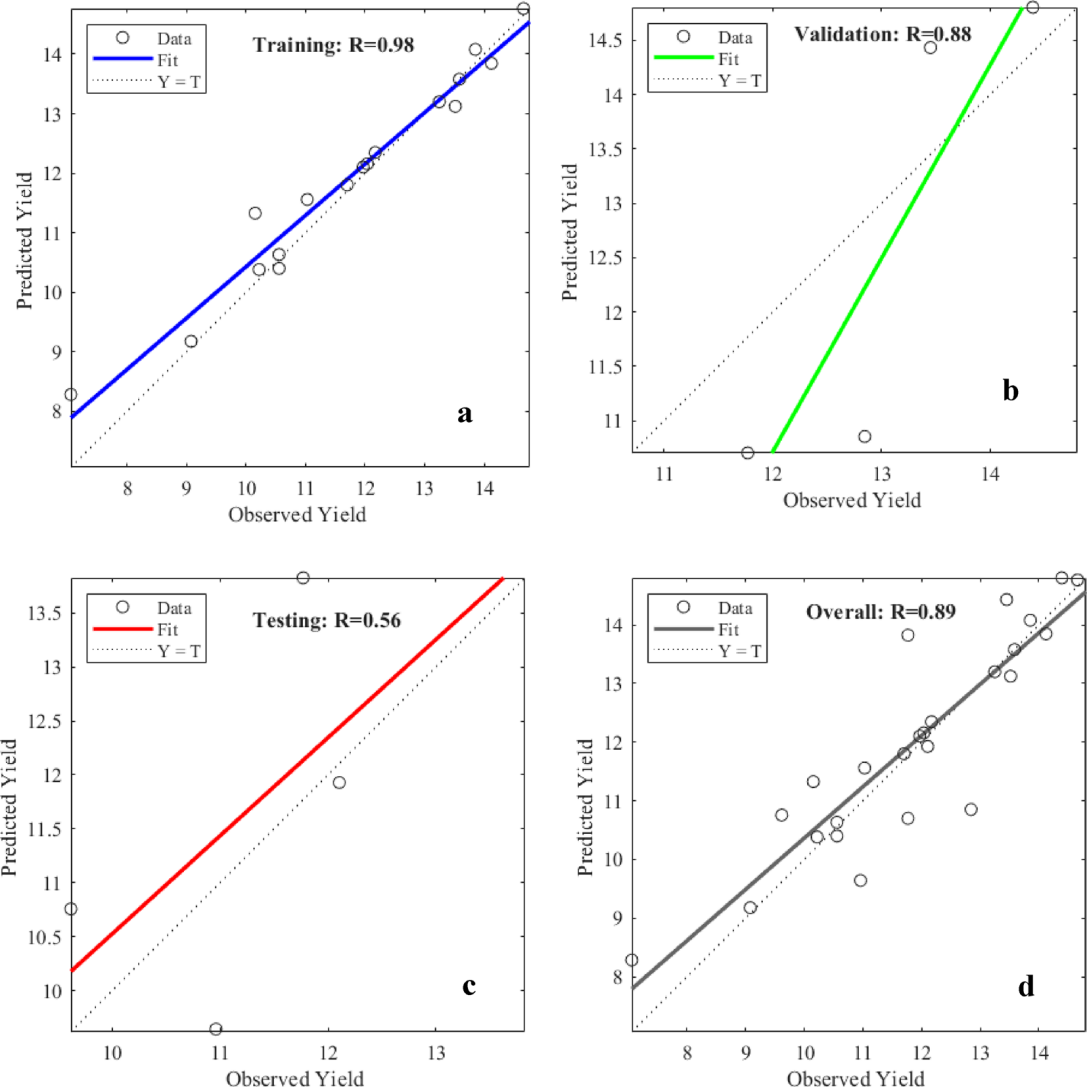
(a-d): Regression Plots of Observed and Estimated Yields of Corn for the ANN Model; a: Training, b: Validation, c: Testing and d: Overall.

In contrast, we employ the linear regression approach to objectively and statistically assess validation results using both observed and estimated data. An additional criterion to assess the effectiveness of the final model is ordinary least square multiple linear regression. The rapid propagation approach was used to train yield predictions using a 9–10–1 network combination. The ANN network showed a significant positive and exceptionally good correlation (*r* = 0.98 at *p* = 0.01) with observed corn yield values in the training phase. Figure 11 shows the regression curve of the ANN model that was used in the present study to forecast the corn yields during the training, validation, and testing phases. The regression plot for corn indicates a strong correlation between predicted and observed yields, with an overall R² value of 0.89, demonstrating the model’s precision. The model achieved R^2^ values of 0.98, 0.88, and 0.56 for training, validation, and testing phases, respectively, indicating consistent and reliable performance across different stages. Ma et al.^53^ predicts the corn yield for the USA with R^2^ value of 0.77 and RMSE value of 1.03 ton/ha by using Bayesian neural network.

**Fig. 12:**
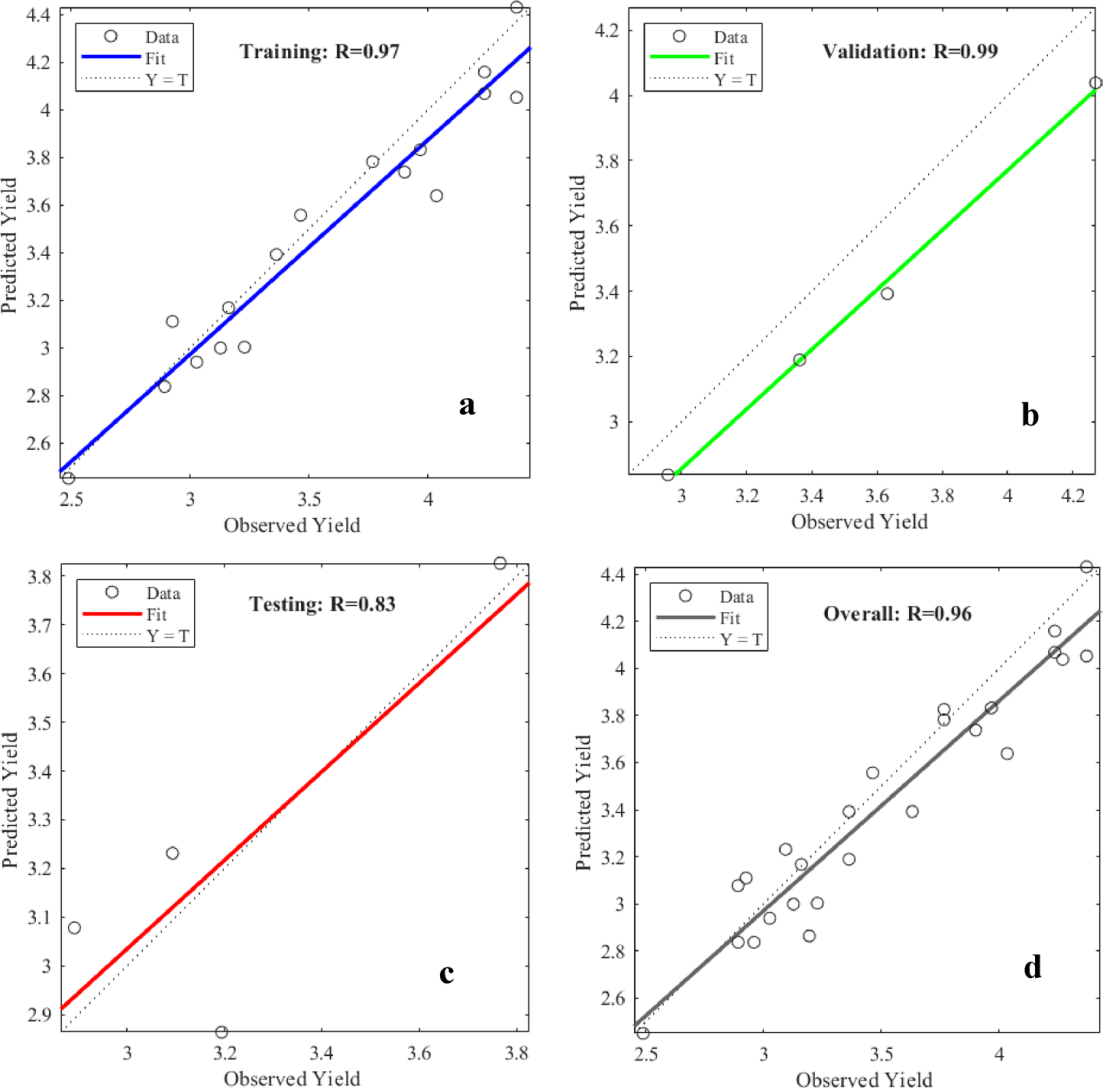
(a-d): Regression Plots of Observed and Estimated Yields of Soybean for the ANN Model; a: Training, b: Validation, c: Testing and d: Overall.

The ANN network showed a significant positive and exceptionally good correlation (*r* = 0.97 at *p* = 0.01) with observed soybean yield values in the training phase. Figure 12 shows the regression plot of the ANN model that was used in the present study to forecast the soybean yields during the training, validation, and testing phases. The regression plot for soybean indicates a strong correlation between predicted and observed yields, with an overall R² value of 0.96, reflecting effective generality by the model. The model achieved R^2^ values of 0.97, 0.99 and 0.83 for training, validation and testing phases respectively, underscoring the model’s capability to predict yields accurately. Najafabadi et al.^54^ predicts the soybean yield in Canada with R^2^ value of 0.76 with RMSE value of 0.225 ton/ha. A comparative analysis indicates that the ANN model demonstrates slightly better performance for soybean than corn, although ANN model achieves satisfactory accuracy for the prediction of both corn and soybean.

The ANN model predicted the corn yield that ranges from 6.81 ton/ha to 15.63 ton/ha, whereas ANN model predicted the soybean yield which ranges from 2.45 ton/ha to 4.43 ton/ha. The maximum predicted corn yield was found in 2014 and the minimum predicted corn yield in 2012. The maximum predicted soybean yield was observed in 2024 while minimum predicted yield of soybean was observed in 2003. The overall robust performance of the ANN models for both crops highlight their potential as reliable tools for yield prediction. These findings can support policymakers and farmers in making informed decisions regarding resource allocation and agricultural planning, ultimately contributing to sustainable agricultural practices.

### Comparison between different models

**Fig. 13:**
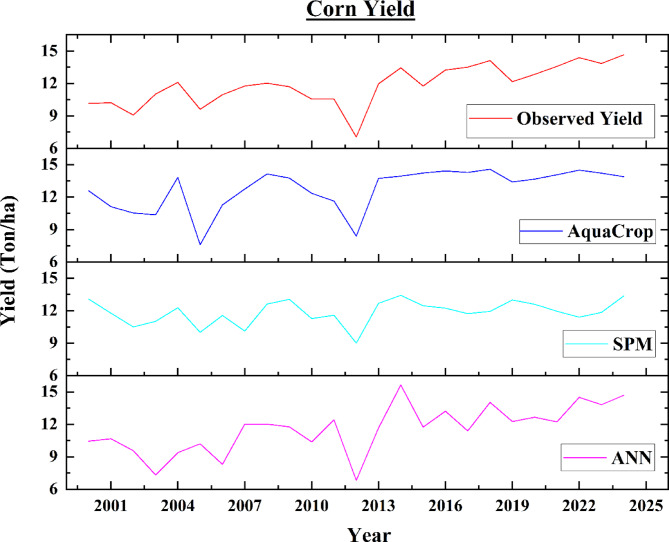
Variation of Observed and estimated yields from different models for corn.

The yield of corn and soybean was estimated by using semi-physical model, AquaCrop model and ANN model for 25-year duration from 2000 to 2024 for Illinois, USA. The ANN model predicted the corn yield to range from 6.81 ton/ha to 15.63 ton/ha and the maximum predicted corn yield was found in 2014 and the minimum predicted corn yield in 2012. The assessed yield of corn by using semi-physical method varied from 9.01 ton/ha to 13.42 ton/ha in which the minimum yield of corn was found in 2012, whereas the maximum yield of corn was found in 2024. Estimated yield of corn by using AquaCrop model ranges from 7.60 ton/ha to 14.42 ton/ha in which the minimum estimated corn yield was found in 2005 and maximum estimated corn yield was found in 2024. The observed yield of corn ranges between 7.06 ton/ha to 14.66 ton/ha in which the minimum and maximum observed yield of corn was observed in 2012 and 2024 respectively. Figure 13 shows the variation of corn observed yield, and estimated yield from different models.

**Fig. 14:**
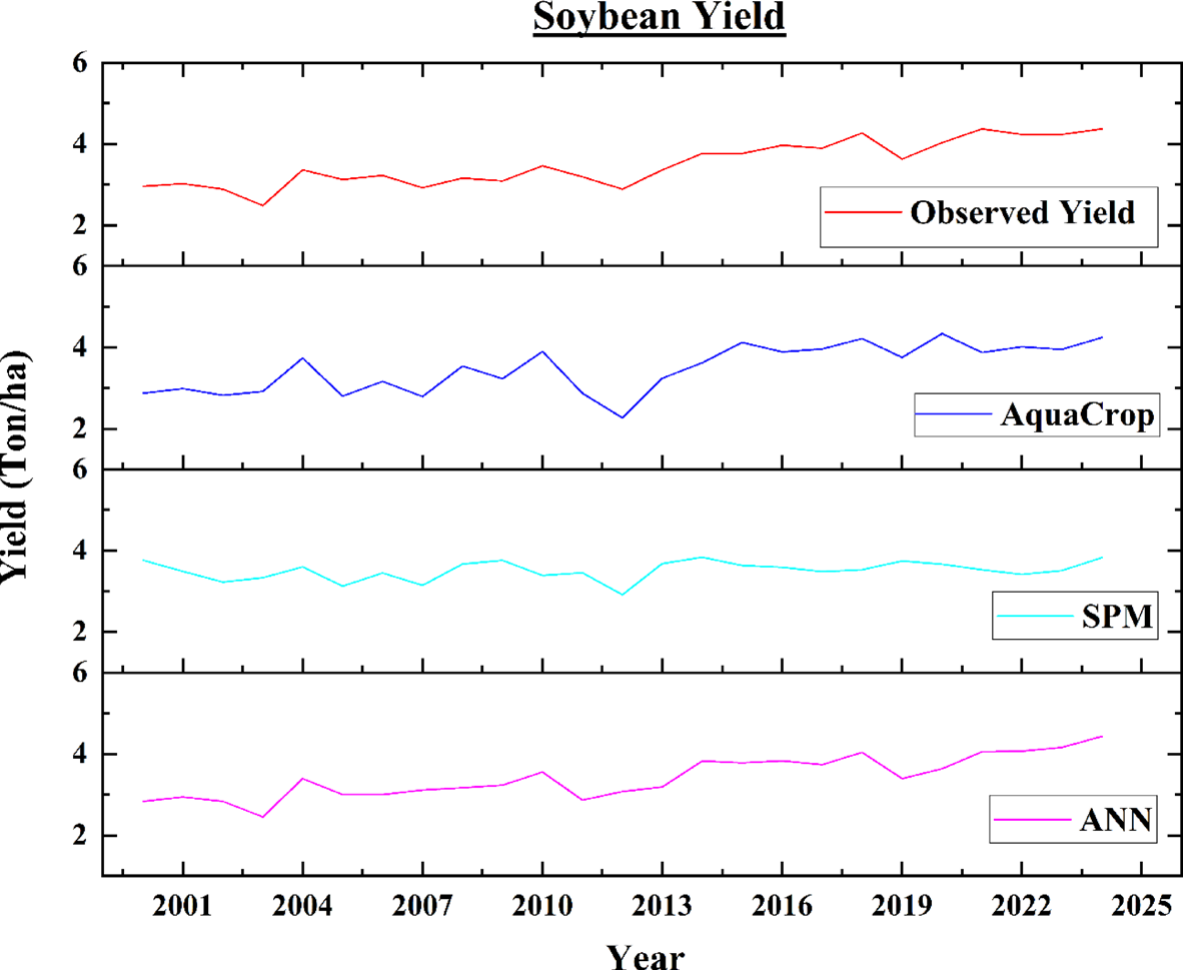
Variation of Observed Yield and Estimated Yields from different models for soybean.

The ANN model predicted the soybean yield which ranges from 2.45 ton/ha to 4.43 ton/ha in which the maximum predicted soybean yield was observed in 2024 while minimum predicted yield of soybean was observed in 2003. The estimated yield of soybeans using semi-physical method ranges from 2.92 ton/ha to 3.84 ton/ha in which the maximum and minimum yield of soybeans were found in 2014 and 2012 respectively. The estimated soybean yields using AquaCrop model range from 2.80 ton/ha to 4.34 ton/ha in which the minimum estimated soybean yield was assessed in 2005, and the maximum estimated soybean yield in 2020, which was very similar to the observed yield of 4.33 ton/Ha. The observed yield of soybean varies from 2.49 ton/ha to 4.37 ton/ha in which the minimum observed yield of soybean was found in 2003 and maximum yield of soybean was found in 2021 and 2024. Figure 14 shows the variation of soybean observed yield, and estimated yields from different models.

The comparison of the performance of AquaCrop model, semi-physical model, and ANN model for corn and soybean yield prediction highlights the higher accuracy of the ANN model. Table 2 depicts the R^2^ and RMSE values for different models. For the estimation of corn yields, ANN achieves the highest R^2^ value of 0.89 and semi-physical model achieves the lowest R^2^ value of 0.60, while ANN model gives the lowest RMSE value of 1.3194 ton/ha and semi-physical model gives the highest RMSE value of 1.4377 ton/ha. But for the estimation of soybean yields, ANN model gives the highest R^2^ value of 0.95 and semi-physical model gives the lowest R^2^ value of 0.42 while ANN model gives the lowest RMSE value of 0.039 ton/ha and semi-physical model gives the highest RMSE value of 0.4888 ton/ha. The ANN model achieved the highest R^2^ and lowest RMSE values for corn and soybeans. This indicated that ANN effectively captures complex, nonlinear relationships in crop yield predictions. High value of R^2^ for the ANN model confirms the accurate prediction of soybean yield. In this study we utilized the ANN models which gives the highest R^2^ values and minimum RMSE for both corn and soybean that indicate the relation between statistical and crop prediction is high interconnected. For better accuracy of AquaCrop we go for ground truth data and experimental data. Accurate forecasting of crop yields before harvest is of paramount importance, particularly in regions prone to the climatic uncertainties.

**Table 2:** Comparison between different models.

Model	Crop	*R* ^2^	RMSE (ton/ha)
AquaCrop	Corn	0.72	1.37
	Soybean	0.77	0.28
SPM	Corn	0.60	1.4377
	Soybean	0.42	0.4888
ANN	Corn	0.89	1.3194
	Soybean	0.96	0.039

## Conclusion

The present study aimed to evaluate the effectiveness of three distinct crop yield prediction models: AquaCrop, semi-physical, and ANN models for predicting corn and soybean yield. The findings demonstrate the advantages and disadvantages of each strategy, showing that semi-physical models offer a compromise between computational efficiency and complexity, whereas AquaCrop offers a process-based understanding of crop reactions to environmental conditions. Conversely, when trained on large, high-quality datasets, ANNs demonstrated a high degree of prediction accuracy. The findings of the present study indicates that no single model is universally superior; rather than model selection should be driven by the availability of input data, computational resources, and the specific goals of the research. For crop climate interactions, AquaCrop is still a useful tool, while ANNs offer plenty of possibility for data-driven forecasting, particularly when there are big and varied datasets available. As an alternative, the semi-physical model is practical and captures important agronomic processes without requiring a lot of calibration. Hybrid modeling techniques that combine the predictive capabilities of ANNs with the mechanistic insights of AquaCrop should be investigated in future studies. Model accuracy may also be increased by integrating data from remote sensing and refining data collection techniques. Further improvements can be achieved by conducting field experiments on agricultural land to validate and refine the model’s predictive abilities. Additionally, early yield prediction facilitates the development of contingency plans by governments to ensure reasonable redistribution of food resources during periods of shortage or scarcity. Farmers, agronomists, and legislators can all make better decisions to maximize agricultural productivity in Illinois and elsewhere by improving these prediction frameworks.

## Data Availability

The datasets used and/or analysed during the current study are available from the corresponding author on reasonable request.
